# A Tapered Cuff Tracheal Tube Decreases the Need for Cuff Pressure Adjustment After Surgical Retraction During Anterior Cervical Spine Surgery: A Randomized Controlled, Double-Blind Trial

**DOI:** 10.3389/fmed.2022.920726

**Published:** 2022-06-29

**Authors:** Yi-Shiuan Li, Elise Chia-Hui Tan, Yueh-Ju Tsai, Mercedes Susan Mandell, Shiang-Suo Huang, Ting-Yun Chiang, Wen-Cheng Huang, Wen-Kuei Chang, Ya-Chun Chu

**Affiliations:** ^1^Department of Anesthesiology, Taipei Veterans General Hospital, Taipei City, Taiwan; ^2^School of Medicine, National Yang Ming Chiao Tung University, Hsinchu, Taiwan; ^3^National Research Institute of Chinese Medicine, Ministry of Health and Welfare, Taipei City, Taiwan; ^4^Institute of Hospital and Health Care Administration, National Yang Ming Chiao Tung University, Hsinchu, Taiwan; ^5^Department of Otorhinolaryngology-Head and Neck Surgery, Taipei Veterans General Hospital, Taipei City, Taiwan; ^6^Department of Anesthesiology, University of Colorado, Aurora, CO, United States; ^7^Department of Anesthesiology, McGovern Medical School, Memorial Hermann-Texas Medical Center, University of Texas Health, Houston, TX, United States; ^8^Department of Pharmacology, Institute of Medicine, Chung Shan Medical University, Taichung, Taiwan; ^9^Department of Pharmacy, Chung Shan Medical University Hospital, Taichung, Taiwan; ^10^Department of Neurosurgery, Neurological Institute, Taipei Veterans General Hospital, Taipei City, Taiwan

**Keywords:** anterior cervical spine surgery, dysphonia, GRBAS, tapered cuff, tracheal tube cuff pressure

## Abstract

**Background:**

Surgical retraction to expose the vertebrae during anterior cervical spine surgery increases tracheal tube cuff pressure and may worsen postoperative sore throat and dysphonia. This randomized double-blind study investigated the effect of cuff shape on intraoperative cuff pressure and postoperative sore throat and dysphonia.

**Methods:**

Eighty patients were randomized to tracheal intubation with a tapered cuff or a conventional cylindrical high-volume low-pressure cuff (control) during anesthesia. Intraoperative cuff pressures were compared. The primary outcome was the incidence of pressure adjustment needed when the cuff pressure increased to > 25 mm Hg after surgical retraction. The secondary outcome was the incidence of postoperative sore throat and dysphonia.

**Results:**

The incidence of pressure adjustment after surgical retraction was significantly lower in the tapered group than in the control group (13% vs. 48%; *P* = 0.001; relative risk reduction, 74%). The median [interquartile range (IQR)] cuff pressure (mm Hg) was significantly lower for the tapered cuff than for the control cuff before surgical retraction [9 (7–12) vs. 12 (10–15); *P* < 0.001] and after retraction [18 (15–23) vs. 25 (18–31); *P* = 0.007]. The median (IQR) postoperative dysphonia score assessed by a single speech-language pathologist was lower in the tapered group than in the control group [4 (3–6) vs. 5.5 (5–7); *P* = 0.008].

**Conclusion:**

A tapered cuff tracheal tube decreased the need for the adjustment of cuff pressure after surgical retraction during anterior cervical spine surgery, thereby avoiding intraoperative pressure increase. It also has a better outcome in terms of dysphonia.

**Clinical Trial Registration:**

[www.clinicaltrials.gov], identifier [NCT04591769].

## Introduction

**FIGURE 1:**
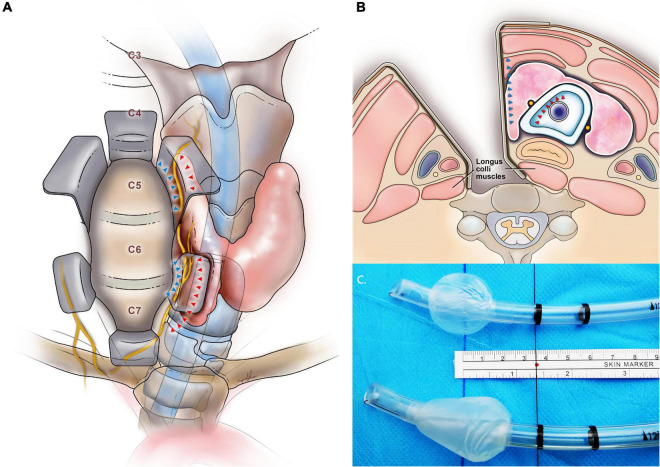
**(A)** The anterior view shows surgical retraction during anterior cervical spine surgery. The retractor displaces the larynx against the unyielding tracheal tube shaft and cuff with compression on the recurrent laryngeal nerve (in yellow). The arrow indicates the force exerted by the retractor blade (blue) and tracheal tube cuff (red). **(B)** Cross-sectional neck anatomy shows the recurrent laryngeal nerve within the medial soft tissue complex between the force from the medial retractor blade (blue) and the tracheal tube cuff (red) when using the right-sided approach (seen from below). **(C)** The tracheal tube cuff shapes and the study groups: cylindrical cuff (i.e., control group, above) and tapered cuff tracheal tube (i.e., tapered group, below).

Sore throat, dysphonia, and dysphagia can occur after neck surgery due to direct surgical injury or prolonged tissue compression (Supplementary Table 1) (1–4). In anterior cervical spine surgery, retractors are used to expose the vertebrae by spreading apart the medial border of the longus colli muscle. As a result, the tracheal tube and surrounding tissues are pulled laterally and compressed (Figure 1A). Compression forces increases the cuff pressure which are then transmitted to the tracheal mucosa and recurrent laryngeal nerve (Figure 1B), thereby increasing the risk of nerve paresis or palsy and subsequent dysphonia (5–7). Investigators reported methods to mitigate compressive forces, including monitoring and limiting the tracheal tube cuff pressure (8), transiently adjusting the cuff pressure by deflating and then reinflating the cuff after retractor placement (6), or use of nasotracheal intubation (9, 10). However, it is not always possible to routinely adjust the cuff pressure after surgical retractor placement because of the proximity of the surgical site to the tracheal tube. Reaching for the tracheal tube could result in contamination of the surgical site or failure to optimize the cuff pressure leading to an accidental air leak from the cuff with a loss of delivered tidal volume.

A tracheal tube with a tapered cuff is designed to minimize longitudinal folds during inflation, improve the tracheal seal, and prevent the leak of secretions and air, even under high airway pressures (Figure 1C). Fluid or air leak was less around a tapered cuff than around a conventional cylindrical high-volume low-pressure cuff (11, 12). Tapered cuffs also achieve a better air seal with reduced cuff pressure (13), thereby leading to a smaller change in cuff pressure during compressive forces such as pneumoperitoneum in laparoscopic surgery (14). There is evidence suggesting tapered cuffs reduce the incidence of postoperative sore throat and dysphonia (15). Based on this evidence, we hypothesized that a tapered cuff may help minimize cuff pressure increases that commonly occur during anterior cervical spine surgery.

Some investigators have reported a larger increase in tapered cuff pressures, compared with that of cylindrical cuffs, after neck extension, rotation, or a change of position in small study cohorts while others have not (16–18). To fully address the effects of cuff shape on pressure after surgical retraction during anterior cervical spine surgery, we conducted a randomized double-blind controlled study using a population sample that was calculated for power of analysis. The primary outcome was the incidence of pressure adjustment needed when the cuff pressure increased to > 25 mm Hg after surgical retraction. The secondary outcome was the incidence of postoperative sore throat and dysphonia.

## Materials and Methods

### Ethics Approval

The study was approved by the Institutional Review Board (approval number: 2020-10-002C, 12 September 2020). All study participants provided written informed consent. The trial was registered before patient enrollment at clinicaltrials.gov (NCT04591769; principal investigator, Ya-Chun Chu; date of registration, 19 October 2020) and conducted in accordance with the Declaration of Helsinki. This report adheres to the Consolidated Standards of Reporting Trials guidelines. This study was designed as a randomized, double-blind, parallel-group trial and conducted at Taipei Veterans General Hospital (Taipei, Taiwan).

### Patient Population

Patients eligible for the study were aged 20–80 years, who were scheduled for elective anterior cervical spine surgery via the right-sided approach (8, 19) by the same surgeon between November 2020 and September 2021. The exclusion criteria were previous trauma to the head and neck area, anticipated difficulty with mask ventilation or tracheal intubation, previous neck surgery, and a history of preoperative hoarseness or vocal cord palsy regardless of etiology, body mass index > 35, and refusal to provide informed consent.

### Randomization and Blinding

Patients were randomly assigned to receive tracheal intubation with a tapered cuff (Shiley TaperGuard Tracheal Tube; Covidien, Mansfield, MA, United States) or a cylindrical cuff (i.e., the control) (Shiley Hi-Contour Tracheal Tube Cuffed; Covidien). Each tracheal tube had an internal diameter of 7.5 mm for men and 7.0 mm for women, unless otherwise specified. Randomization was performed using a computer-generated list in blocks of four in a 1:1 ratio by a statistician. Group allocation was unknown by the intubating anesthesiologist until immediately before tracheal intubation. After intubation, another investigator, blinded to group allocation, inflated the pilot balloon with room air through a three-way stopcock attached to an extension that was accessible at the foot of the bed. The investigator that collected cuff pressure data and a speech-language pathologist who assessed voice quality were blinded to group allocation. The intubating anesthesiologist was responsible for removing the tracheal tube at the conclusion of surgery.

### Conduct of the Study

Anesthesia was induced by using propofol (1.5–2.5 mg kg^–1^), fentanyl (3 μg kg^–1^), and cisatracurium (0.2 mg kg^–1^). Tracheal intubation was performed using the GlideScope Titanium Reusable System with a LoPro blade (GlideScope Video Monitor; Verathon Medical, Burnaby, BC, Canada) after 5 min of mask ventilation when complete neuromuscular blockade was confirmed by a zero train of four counts. The vocal cords were visualized between two black line markings (2 cm apart) proximal to the cuff; the proximal line was 3.5 cm from the middle of the cuff (Figure 1C). After patient positioning, the tip of the tracheal tube was also identified and adjusted to thoracic vertebral level 2 (T2)–T4 during fluoroscopic visualization by the surgeon. The tracheal tube was then secured with tape at the left angle of the mouth. The pilot balloon of the cuff was connected to a disposable pressure transducer system (DTXPlus; Argon Medical Systems, Yishun, Singapore). The cuff pressure was continually displayed on the patient monitor (Infinity Kappa; Draeger Medical Systems, Andover, MA, United States). A three-way stopcock was used to adjust the amount of air in the cuff. The cuff was initially inflated with 2 mL of air and then, in stepwise increments of 0.5 mL, air were injected until the following three conditions were met: (1) no air leak was identified by auscultation using a stethoscope over the sternal notch; (2) the measured expired tidal volume was within the 95% limit of the predetermined setting on the ventilator; and (3) no alarm occurred indicating inadequate mechanical ventilation when the fresh gas flow was lowered to 0.5 L min^–1^ (i.e., low-flow anesthesia) for 3 min. Ventilation was set in volume-controlled auto-flow mode (Dräger Medical GmbH, Lübeck, Germany) at a flow rate of 1.2 L min^–1^, a tidal volume of 6–8 mL kg^–1^ of ideal body weight, an inspiratory-to-expiratory ratio of 1:2, and a positive end-expiratory pressure of 5 cm H_2_O to maintain an end-tidal pCO_2_ of 35–40 mm Hg and a peak airway pressure of < 20 cm H_2_O. The cuff pressure was checked for the presence of a leak after neck extension and recorded as the baseline pressure before surgical retraction.

The maximal cuff pressure was recorded after final positioning of the surgical retractors. If the maximal pressure was > 25 mm Hg (9, 20), then 0.5 mL of air was aspirated in a stepwise manner until reaching a pressure of ≤ 25 mm Hg. Anesthesia was maintained using an oxygen-sevoflurane mixture and intermittent boluses of cisatracurium were given intravenously to maintain a train of four counts of ≤ 3. After removing the retractors, we recorded whether an air leak existed. At the end of surgery, the trachea and pharynx were carefully suctioned while the patient was anesthetized. Neuromuscular blockade was reversed with neostigmine (40 μg kg^–1^) once the train of four count was 4. After ensuring adequate neuromuscular reversal, the inhalational anesthesia was stopped. The patient was allowed to awaken spontaneously without stimulation. The tracheal tube was then removed when the patient regained consciousness and fully recovered from the neuromuscular blockade with a train of four ratio ≥ 90%.

Two hours after surgery and on postoperative day 1, the patients were asked to assess throat soreness by using a 10-point numeric rating scale. The assessment was conducted by research personnel blinded to group allocation. Hoarseness was assessed using a grading system previously established in clinical studies where: “0” was no impairment; “1,” was clinically detectable and “2” was severe (9, 10, 21). Five characteristics of the voice used for rating dysphonia adhered to the GRBAS scale and included the Grade of vocal impairment, Roughness, Breathiness, Asthenia (physical weakness of voice), and Strain of the voice (22–24). The speech-language pathologist, who was blinded to group allocation, calculated the GRBAS scores from the voice recordings. Each GRBAS component was rated on a four-point integer scale as previously described: “0” was normal; “1,” mild impairment; “2,” moderate impairment; and “3,” severe impairment. The total score was recorded, as previously described (25, 26).

### Primary Outcomes

Intraoperative cuff pressures were compared at five timepoints: after (1) the initial seal for tracheal intubation, (2) neck extension, (3) surgical retraction, (4) pressure adjustment, and (5) removal of the retractors. The primary outcome was the incidence of pressure adjustment needed when the cuff pressure increased to > 25 mm Hg (34 cm H_2_O) after surgical retraction. We chose a pressure of > 25 mm Hg as a benchmark of identifying post-retraction pressures that could contribute to postoperative complications based on the findings of a previous endoscopic study (27). The study showed a normal caliber of tracheal mucosal blood vessels at cuff pressure of 22 mm Hg (30 cm H_2_O). The vessel caliber was partially occluded at pressures of 29 mm Hg (39 cm H_2_O). We therefore took the mean pressure between the two clinical correlates (22 and 29 mm Hg) as 3.5 and rounded down to a whole integer of 3 which resulted in a pressure of 25 mm Hg (22 + 3 mm Hg). This estimated pressure was below the threshold of 29 mm Hg where mucosal blood flow was impaired and did not result in an air leak from the tracheal cuff (9, 10, 20).

### Sample Size Calculation

We estimated the risk of a cuff pressure increase of > 25 mm Hg after retraction at approximately 60% for cylindrical tracheal cuffs (9, 10), and estimated that tapered cuffs would reduce this risk to 30%. We determined that a sample size of 40 patients per group would be needed at a two-sided significance level of 0.05 (α = 0.05) and 80% power (β = 0.2). Furthermore, the estimated power of all participants (*n* = 80) and surgical level subgroups (above the C6/7 level [*n* = 44] and at the C6/7–T1 level [*n* = 36]) was > 0.8.

### Statistical Analysis

Sample distributions were evaluated using the Kolmogorov–Smirnov test to evaluate the normality of the data. Continuous data derived from demographic characteristics were compared using the Mann–Whitney *U* test. Categorical data were compared using the chi square test or Fisher’s exact test. Data are summarized as the median (25th–75th percentile interquartile range [IQR]) or as the number (%), as appropriate.

Factors associated with a cuff pressure > 25 mm Hg after surgical retraction were analyzed using a binary logistic regression model. Risk estimates were calculated for the odds ratio and the 95% confidence interval (CI). The primary outcome, the incidence of pressure adjustment after surgical retraction, was compared using the chi square test. Absolute and relative risk reductions for pressure adjustment after surgical retraction were calculated for all study participants and subpopulations.

Cuff pressures at the five timepoints were compared between study groups by using a generalized estimating equation (GEE) model with unstructured correlation, with baseline characteristics, treatment group, time, and initial cuff pressure as the fixed effects, and study participants as the random effect. We recognized that cuff pressure data may not all fit normal distribution. The GEE approach is a marginal model commonly used for longitudinal/clustered data analysis in clinical trials. It is also robust for non-normally distributed data in the event that the distribution of cuff pressure data were non-parametric (28–30). We also analyzed the time × treatment interaction by using the difference-in-differences regression method to examine the pre–post change in cuff pressure at each observed timepoint to delineate the effect of the group on surgical intervention Data are summarized as the median (IQR) and shown as the mean (standard error of mean) (31). Postoperative outcomes were compared between groups using GEE models. All statistical analyses were conducted using SAS, version 9.4 (SAS Institute Inc., Cary, NC, United States). Two-sided *P*-values < 0.05 were statistically significant.

## Results

### Demographic Data

**FIGURE 2:**
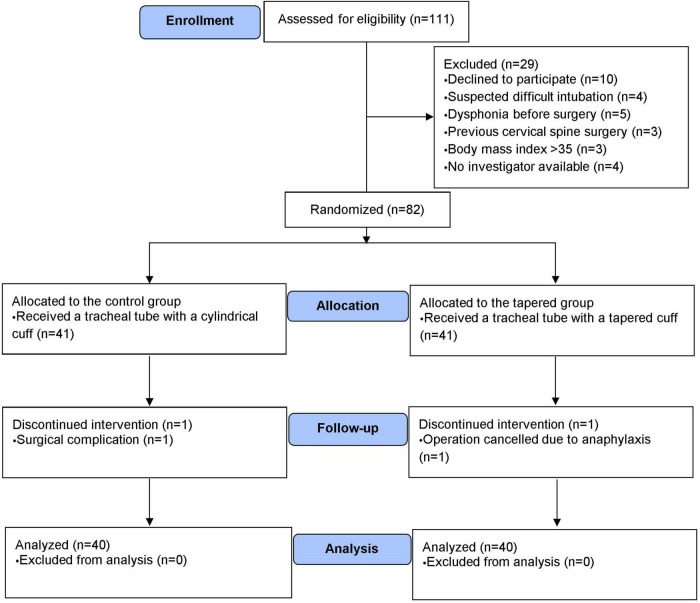
The CONSORT diagram shows the patient recruitment process. CONSORT, Consolidated Standards of Reporting Trials.

Eighty-two patients were included in the study. Eighty patients completed the study and were included in the analysis (Figure 2). Clinical and surgical characteristics were comparable between the groups (Table 1).

**TABLE 1:** Patients’ characteristics and surgical data.

	Control group		Tapered group	
Study participants, n	40		40	
Age (y), median (IQR)	61	(48–69)	57	(46–64)
Male sex, n (%)	24	(60)	24	(60)
Body mass index, median (IQR)	26	(23.6–27.8)	26	(24.0–26.7)
Smoking habit, n (%)	5	(13)	7	(18)
**ASA physical status n (%)**				
I	11	(28)	11	(27)
II	28	(70)	28	(70)
III	1	(3)	1	(3)
**Surgical characteristics**				
**Surgery, based on instrumentation, n (%)**				
Cervical disc arthroplasty	20	(50)	22	(55)
Discectomy and fusion	10	(25)	10	(25)
Corpectomy and fusion	5	(13)	1	(3)
Combined	5	(13)	7	(18)
Level operated on, median (IQR)	2	(1–3)	2	(2–3)
**Surgical level, n (%)**				
above C6/7	21	(53)	23	(58)
at C6/7–T1	19	(48)	17	(43)
**Duration (min), median (IQR)**				
Surgery	155	(135–214)	155	(125–214)
Surgical retraction	100	(71–158)	97	(76–151)
Tracheal intubation	231	(186–287)	215	(188–292)
GRBAS dysphonia score, median (IQR) Total score, preoperative	4	(3–4)	4	(3–4)

*ASA, American Society of Anesthesiologists. The interquartile range (IQR) is the 25th–75th percentiles.*

### Intraoperative Cuff Pressure and the Need for Pressure Adjustment After Surgical Retraction

The incidence of pressure adjustment after surgical retraction was significantly lower in the tapered than the control group (13 vs. 48%, *P* = 0.001; Table 2). A surgical level at cervical vertebra 6/7 (C6/7)–T1 was independently associated with an increased risk of pressures > 25 mm Hg after surgical retraction (adjusted odds ratio, 13.1; 95% CI, 2.4–72.7; *P* = 0.003, vs. the level above C6/7; Table 2). The use of the tapered cuff tube was associated with a reduced risk (adjusted odds ratio, 0.08; 95% CI, 0.02–0.4; *P* = 0.002, vs. the control; Table 2). The primary outcome, pressure adjustments after surgical retraction were fewer with tapered cuffs than control in all study participants, regardless of whether the surgical level was at C6/7–T1 or above C6/7 (Table 2). The relative risk reduction was 74% (95% CI, 36–89) for all study patients; 100% for patients with a surgical level above C6/7, and 60% (95% CI, 13–82) for patients with a surgical level at C6/7–T1 (Table 3).

**TABLE 2:** Factors associated with maximal cuff pressure > 25 mmHg after the retractors splayed.

Variable	Comparison	Univariable			Multivariable		
			
		OR	(95% CI)	*P*-value	OR	(95% CI)	*P*-value
Tapered cuff	Control	0.16	(0.05–1.09)	0.001	0.08	(0.02–0.40)	0.002
Surgical levels: including C6/7–T1	above C6/7	8.72	(2.79–27.20)	<0.001	13.12	(2.37–72.66)	0.003
Cuff pressure before retraction	+1	1.06	(0.93–1.21)	0.367	1.01	(0.84–1.22)	0.899
Age	+1	1.04	(1.00–1.09)	0.068	1.01	(0.95–1.07)	0.790
Male sex	Female	1.16	(0.44–3.10)	0.765	0.98	(0.25–3.88)	0.979
BMI	+1	1.13	(0.97–1.33)	0.122	1.07	(0.85–1.34)	0.554
Smoking habit	none	0.18	(0.02–1.46)	0.108	0.10	(0.01–1.39)	0.086
No. of surgical levels	+1	2.05	(1.20–3.49)	0.009	1.24	(0.54–2.83)	0.618

*CI, confidence interval; OR, odds ratio.*

**TABLE 3:** The incidence of pressure adjustment when the cuff pressure increased to > 25 mmHg after surgical retraction.

Study population n (%)	Control group	Tapered group	*P*-value	Absolute risk reduction	Relative risk reduction	Number needed to treat
				% (95% CI)	% (95% CI)	n (95% CI)
All study participants, *n* = 80 (100%)	(*n* = 40)	(*n* = 40)				
>25 mm Hg, n (%)	19 (48)	5 (13)	0.001	35 (16–54)	74 (36–89)	3 (2–7)
Surgical level above C6/7, *n* = 44 (55%)	(*n* = 21)	(*n* = 23)				
>25 mm Hg, n (%)	5 (24)	0 (0)	0.019	24 (6–42)	100	5 (3–18)
Surgical level at C6/7–T1, *n* = 36 (45%)	(*n* = 19)	(*n* = 17)				
>25 mm Hg, n (%)	14 (74)	5 (29)	0.018	44 (15–74)	60 (13–82)	3 (2–7)

*CI, confidence interval.*

**FIGURE 3:**
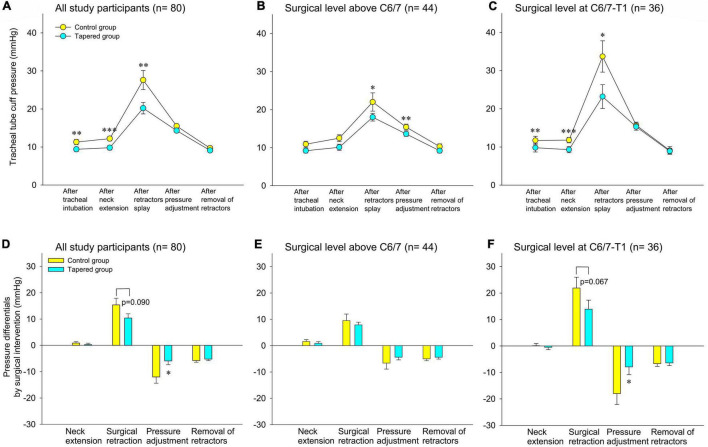
Tracheal tube cuff pressure during surgery **(A–C)** and pressure differentials by surgical intervention **(D–F)**. **(A,C)** All study participants. **(B,D)** Surgical levels above C6/7. **(C,F)** Surgical level at C6/7–T1. Data are presented as the mean and the standard error of the mean, *P < 0.05, ^**^P < 0.01, and ^***^P < 0.001.1 mm Hg = 1.36 cm H_2_O.

Supplementary Table 2 shows the intraoperative cuff pressures. The median (IQR) cuff pressures (mm Hg) were significantly lower for the tapered cuff than for the control cuff after tracheal intubation [9 (7–12) vs. 11 (8–14); *P* = 0.009)], after neck extension [9 (7–12) vs. 12 (10–15); *P* < 0.001] and after retraction [18 (15–23) vs. 25 (18–31); *P* = 0.007, Figure 3A]. Pressure differentials (i.e., pre–post change) caused by surgical retraction and pressure adjustment were smaller in the tapered group than in the control group (Figure 3D).

### Subpopulation Analysis According to the Surgical Level Treated

The patients were dichotomized into two groups for further analysis based on whether the surgery was at the C6/7–T1 level or above the C6/7 level. Post-retraction cuff pressures were significantly lower in the tapered group compared to the controls when the surgical level was at C6/7–T1 (Figure 3C), but not when the level was above C6/7 (Figure 3B). Cuff pressure differentials by surgical retraction and pressure adjustment were also less with the tapered cuff than with the control cuff at a surgical level of C6/7–T1 (Figure 3F), but not when the level was above C6/7 (Figure 3E).

### Postoperative Outcomes

No significant differences were found between the two study groups in the severity of postoperative sore throat and self-assessed hoarseness (Table 4). However, the median (IQR) GRBAS dysphonia score was significantly lower in the tapered group than in the control group on postoperative day 1 [4 (3–6) vs. 5.5 (5–7); *P* = 0.008, Table 4].

**TABLE 4:** Postoperative sore throat and dysphonia.

	Control group	Tapered cuff group	*P-*value
**Sore throat, median (IQR)**			
NRS, 2 h after surgery	5 3–8)	5 (3–7)	0.964
NRS, postoperative day 1	3 (1–5)	3.5 (1.5–5)	0.574
**Self-assessed hoarseness, postoperative day 1, n (%)**			
None	9 (23)	14 (35)	0.324
Obvious	22 55)	21 (53)	
Severe	9 (23)	5 (13)	
**GRBAS dysphonia score, median (IQR)**			
Total score, postoperative day 1	5.5 (5–7)	4 (3–6)	0.008

*The cuff pressure is controlled and set at the pressure > 25 mmHg after the retractors were set up. The interquartile range (IQR) is the 25th–75th percentiles. GRBAS, grade, roughness, breathiness, asthenia, and strain for dysphonia; NRS, numeric rating scale.*

## Discussion

Our study demonstrated that cuff pressures during anterior cervical spine surgery were lower with a tapered cuff than a cylindrical cuff. Lower pressures were observed for the just-seal pressure before surgical retraction and the maximal pressure after retraction. These findings were influenced by the cervical level of the surgical treatment: pressure increases were more frequent at C6/7–T1 in our surgical population. Tracheal tubes with a tapered cuff needed less pressure adjustment under all study conditions. Postoperative dysphonia scores were lower in the tapered group than in the control group, even when the cuff pressure was controlled and set at ≤ 25 mm Hg for both groups. We conclude that the tapered cuff design had the beneficial effect of decreasing the need for cuff pressure adjustment after surgical retraction, and of achieving a better immediate outcome of voice quality. Our results discovered the tapered cuff tracheal tube as an alternative to conventional cylindrical cuffs for neck surgery when intraoperative cuff adjustment is not feasible.

Attempts to adjust cuff pressures during surgery can cause accidental loss of occlusion pressure and increase the risk of inadvertent air leaks (32, 33). We needed fewer pressure adjustments when using tapered cuffs during anterior cervical spine surgery. This suggests that tapered cuffs may accommodate changes in compressive forces more readily than cylindrical cuffs. Overall, this appears to offer greater safety by reducing the need for pressure adjustments and the consequent complications of over and under-inflation.

An explanation for this advantage is that the tapered cuff is designed to minimize longitudinal folds, which can be the source of air leaks and aspiration of secretions (11, 34). Our findings support previous observations (35); we found that tapered cuffs had a lower sealing pressure. The sealing pressures in our study were lower than the cuff pressure of 20–30 cm H_2_O (14.7–22.1 mm Hg) commonly used in clinical practice. The median occlusion cuff pressures of 9 mm Hg for tapered cuffs and 11 mm Hg for cylindrical cuffs, needed for a leak-free seal, were higher than the pressures found in a viscoelastic model of the trachea (36). The model predicted that cuffs with different designs required a pressure of only 8.8 mm Hg (12 cm H_2_O) for a complete air seal (36); the findings for tapered cuffs in this study are consistent with predicted values from simulated models (36). Further, the safety of our occlusion pressures was confirmed in our human study participants.

The baseline median pressure of approximately 10 mm Hg (13.6 cm H_2_O) and the maximal pressure of 25 mm Hg (34 cm H_2_O) chosen for adjustment, made the allowable pressure range approximately 15 mm Hg (25 minus 10 mm Hg). The median pressure difference of 7 mm Hg after surgical retraction between our study groups accounted for one-half of the range. While continuous cuff pressure monitoring and adjustment is a recommended approach for reducing pressure-related complications (32), our data indicates that use of a tapered cuff confers additional safety. This is particularly true when continuous monitoring is not available. Further, the pilot balloon of the tube is not always easily accessible and physical impediments may delay or prevent appropriate monitoring. Our data supports the use of the just-seal pressure as the baseline for the tapered cuff tube to reach minimal occlusion pressure and potentially reduce the need for pressure adjustment.

Our observations that baseline pressures were significantly lower in the tapered than control group suggested that the use of “just-sealed” pressure is a potential safety measure that can independently reduce the risk of mucosal and nerve compression. The greater differential for pressure measurements between the baseline just-sealed pressure and the target pressure of 25 mm Hg supports our impression of the improved safety margin for tapered cuffs.

Other investigators have reported a larger increase in tapered cuff pressures than in cylindrical cuff pressures after neck extension, rotation, or change of position (16–18). Differences in study design, including the site or type of surgery and the selection of baseline pressures, likely explain the unique findings of different studies. For example, some studies used a baseline pressure of 15 mm Hg (20 cm H_2_O) for all study patients (16–18), regardless of the sealing pressure determined by clinical auscultation, whereas we used the just-seal pressure for every patient.

Previous studies reported a greater risk of postoperative vocal cord palsy in patients who have surgery at the C6/7–T1 level (6, 37, 38). The risk of postoperative vocal cord palsy can be related to the cuff pressure when surgical site levels vary. However, to date, no published reports exist on the influence of spinal level on cuff pressure after tissue retraction. In this study, we dichotomized our patient population based on the surgical level and found that the surgical level influenced the increase in cuff pressure. The risk of higher pressures (>25 mm Hg) was 13-fold higher at the C6/7–T1 level than for levels above C6/7. Our observation of cuff pressure increases by surgical level coincided with the levels with higher risk of postoperative vocal cord palsy reported in previous studies (6, 37, 38). In clinical practice, surgery may involve multiple levels, especially with instrumentation spanning the upper and lower levels of the cervical spine. Nevertheless, the benefit of the tapered cuff tube was demonstrated by significant risk reduction in both subgroups.

This study had limitations. We did not include a group with cuff pressures > 25 mm Hg after retraction because of safety concerns. Therefore, we cannot hypothesize about possible postoperative outcomes for pressures greater than our target. The study findings are specific for anterior cervical spine surgery. We did not include patients who underwent alternate surgeries to test our study design for external validity and cannot determine whether our findings could be representative of tapered cuff performance in other types of surgery that require pneumoperitoneum or in critically ill patients on long-term mechanical ventilation. The study findings are specific for anterior cervical spine surgery when using tracheal tubes with an internal diameter of 7.0 mm for females and 7.5 mm for males during general anesthesia with neuromuscular relaxation. Further, the better outcomes of using tapered cuff tube in our study cannot be generalized to other potential airway complications that have been reported after anterior cervical surgery. This study was conducted by a single surgeon at one center. Further testing is needed to determine the external validity of our findings.

In conclusion, tapered cuffs required fewer intraoperative pressure adjustments and produced better postoperative voice outcomes in our randomized double-blind study of anterior cervical spinal surgery. Tapered cuffs may confer improved patient outcomes if continuous cuff pressure monitoring is impossible or if the access for pressure adjustment is difficult.

## Implication for Practice and Research

Surgical retraction to expose the vertebrae during anterior cervical spine surgery increases tracheal tube cuff pressure and may worsen postoperative sore throat and dysphonia. Limiting or adjusting cuff pressure after surgical retraction reduces the incidence of postoperative sore throat and dysphonia but is not always possible to routinely performed the proximity of the surgical site to the tracheal tube or pressure monitoring is unavailable. Our prospective, randomized controlled, double-blind study revealed a tapered cuff tracheal tube, compared with a conventional cylindrical high-volume low-pressure cuff tube, decreased the need for the adjustment of cuff pressure after surgical retraction during anterior cervical spine surgery, thereby avoiding intraoperative pressure increase. It also has a better outcome in terms of dysphonia.

## Data Availability Statement

The original contributions presented in this study are included in the article/Supplementary Material, further inquiries can be directed to the corresponding author.

## Ethics Statement

The studies involving human participants were reviewed and approved by the Institutional Review Board of Taipei Veterans General Hospital (approval number: 2020-10-002C, 12 September 2020). The patients/participants provided their written informed consent to participate in this study.

## Author Contributions

Y-SL helped recruit the patients, conduct the trial, collect the data, and draft the manuscript. EC-HT helped design the study, analyze and interpret the data, and draft the manuscript. Y-JT helped conduct the trial and collect and analyze the data. MSM helped interpret the data and draft the manuscript. S-SH helped study design and analyze and interpret the data. T-YC helped recruit the patients, conduct the trial, and collect the data. W-CH helped study design, recruit the patients, conduct the trial, and interpret the data. W-KC helped study, conduct the trial, and interpret the data. Y-CC helped design the study, recruit the patients, conduct the trial, collect and analyze the data, and draft. All authors edited the draft, revised, and approved the manuscript.

## Conflict of Interest

The authors declare that the research was conducted in the absence of any commercial or financial relationships that could be construed as a potential conflict of interest.

## Publisher’s Note

All claims expressed in this article are solely those of the authors and do not necessarily represent those of their affiliated organizations, or those of the publisher, the editors and the reviewers. Any product that may be evaluated in this article, or claim that may be made by its manufacturer, is not guaranteed or endorsed by the publisher.
