# Supplementary material for: A Tapered Cuff Tracheal Tube Decreases the Need for Cuff Pressure Adjustment After Surgical Retraction During Anterior Cervical Spine Surgery: A Randomized Controlled, Double-Blind Trial
Source: Front Med (Lausanne). 2022 Jun 29;9:920726. doi: 10.3389/fmed.2022.920726 (PMC9276934; doi:10.3389/fmed.2022.920726)
Supplement: Supplementary file 1 [file Table_1.docx]

Supplementary table S1. Reported complication rates and ranges after anterior cervical spine surgery in reviews

|  | Incidence (%) | |
| --- | --- | --- |
|  | Epstein (39), 2019 | Yee (40), 2020 |
| Recurrent laryngeal nerve palsy | 0.9–3.1 | 1.3 (0.1–60.9) |
| Acute | 0.9–8.3 |  |
| Chronic | 2.5 |  |
| Dysphagia |  | 5.3 (0.2–87.5) |
| Immediate | 1.7–67 |  |
| Delayed | 1.7–9.5 |  |
| Postoperative hematoma | 1.3–5.6 | 1.0 (0–12.5) |
| Adjacent segment disease | N/A | 8.1 (0.9–52.2) |
| Pseudoarthrosis | N/A | 2.0 (0–55.0) |
| Instrumentation failure | 0.1–0.9 | 2.1 (0–50.0) |
| New/worsening myelopathy | 0.2–3.3 | 0.5 (0–25.7) |
| New/worsening radiculopathy | 1.3 | 3.0 (0.1–7.7) |
| Cerebrospinal fluid leak | 0.5–1.7 | 0.5 (0.03–7.7) |
| Wound infection | 0.9–1.6 | 1.2 (0–16.7) |
| Horner's syndrome | 0.06–1.1 | 0.4 (0.1–2.5) |
| Esophageal perforation | 0.3–0.9 | 0.2 (0–0.46) |
| Vertebral artery injury | N/A | 0.4 (0.2–2.2) |
| Respiratory insufficiency | 1.1 | N/A |

NA, not available.

References:

1. Epstein NE. A review of complication rates for anterior cervical diskectomy and fusion (ACDF). *Surg Neurol Int*. (2019) 10:100. doi: 10.25259/SNI-191-2019
2. Yee TJ, Swong K, Park P. Complications of anterior cervical spine surgery: a systematic review of the literature. *J Spine Surg.* (2020) 6:302-22. doi: 10.21037/jss.2020.01.14
