# Supplementary material for: A Tapered Cuff Tracheal Tube Decreases the Need for Cuff Pressure Adjustment After Surgical Retraction During Anterior Cervical Spine Surgery: A Randomized Controlled, Double-Blind Trial
Source: Front Med (Lausanne). 2022 Jun 29;9:920726. doi: 10.3389/fmed.2022.920726 (PMC9276934; doi:10.3389/fmed.2022.920726)
Supplement: Supplementary file 2 [file Table_2.DOCX]

Supplementary table S2. Intraoperative endotracheal tube cuff pressures and pressure differentials by surgical intervention

|  | Control group | | Taper group | | Adjusted difference  (95% CI) | | *P* value |
| --- | --- | --- | --- | --- | --- | --- | --- |
|  | median | (IQR) | median | (IQR) |  |  |  |
| **All participants (n=80)** | | | | | | | |
| Cuff pressure, mm Hg |  |  |  |  |  |  |  |
| After tracheal intubation | 11 | (8, 14) | 9 | (7, 12) | -2.0 | (-3.5, -0.5) | 0.009 |
| After neck extension | 12 | (10, 15) | 9 | (7, 12) | -2.7 | (-4.1, -1.3) | <0.001 |
| After retractor splay | 25 | (18, 31) | 18 | (15, 23) | -7.7 | (-13.2, -2.1) | 0.007 |
| After pressure adjustment | 16 | (13, 19) | 15 | (12, 17) | -1.5 | (-3.2, 0.1) | 0.065 |
| After removal of retractor | 10 | (7, 12) | 9 | (7, 11) | -0.6 | (-2.0, 0.7) | 0.354 |
| Pressure differential by surgical intervention, mmHg | | | | | | | |
| Neck extension | 1 | (-1, 3) | 0 | (-2, 3) | -0.7 | (-2.1, 0.72) | 0.342 |
| Surgical retraction | 13 | (7, 17) | 9 | (5, 12) | -5.0 | (-10.8, 0.8) | 0.090 |
| Pressure adjustment | -10 | (-15, 0) | 0 | (-10, 0) | 6.1 | (0.7, 11.6) | 0.029 |
| Removal of retractors | -6 | (-9, -3) | -5 | (-8, -2) | 0.90 | (-0.8, 2.6) | 0.307 |
| **Surgical levels: above C6/7, n= 44** | | | | | | | |
| Cuff pressure, mm Hg |  |  |  |  |  |  |  |
| After tracheal intubation | 11 | (8, 13) | 10 | (7, 10) | -1.2 | (-3.6, 1.1) | 0.309 |
| After neck extension | 12 | (11, 15) | 9 | (7, 11) | -2.1 | (-4.5, 0.32) | 0.090 |
| After retractor splay | 19 | (15, 25) | 18 | (15, 22) | -4.9 | (-9.5, -0.3) | 0.037 |
| After pressure adjustment | 15 | (13, 19) | 14 | (11, 17) | -3.0 | (-5.0, -1.0) | 0.003 |
| After removal of retractor | 10 | (8, 12) | 9 | (7, 11) | -0.9 | (-2.6, 0.8) | 0.296 |
| Pressure differential by surgical intervention, mmHg | | | | | | | |
| Neck extension | 1 | (1, 4) | 0.0 | (-1, 3) | -0.9 | (-2.8, 1.1) | 0.395 |
| Surgical retraction | 8 | (1, 13) | 9.0 | (5, 11) | -2.8 | (-7.0, 1.3) | 0.181 |
| Pressure adjustment | 0 | (-11, 0) | 0.0 | (-9, 0) | 1.9 | (-2.7, 6.4) | 0.426 |
| Removal of retractors | -4 | (-8, -3) | -4.0 | (-8, -1) | 2.1 | (0.2, 4.1) | 0.035 |
| **Surgical levels: at C6/7**–**T1, n= 36,** | | | | | | | |
| Cuff pressure, mmHg |  |  |  |  |  |  |  |
| After tracheal intubation | 10 | (8, 14) | 9.0 | (7, 12) | -2.4 | (-4.2, -0.6) | 0.010 |
| After neck extension | 12 | (10, 14) | 8.0 | (7, 12) | -3.0 | (-4.5, -1.5) | <.0001 |
| After retractor splay | 29 | (25, 35) | 17 | (15, 31) | -12.1 | (-21.4, -2.8) | 0.011 |
| After pressure adjustment | 16 | (13, 19) | 16 | (12, 18) | -0.8 | (-3.1, 1.5) | 0.470 |
| After removal of retractor | 9 | (6, 11) | 8 | (7, 10) | 0.3 | (-2.0, 2.5) | 0.813 |
| Pressure differential by surgical intervention, mmHg | | | | | | | |
| Neck extension | 1 | (-2, 3) | 0 | (-3, 2) | -0.6 | (-2.7, 1.5) | 0.573 |
| Surgical retraction | 16 | (12, 27) | 8 | (4, 23) | -9.1 | (-18.9, 0.6) | 0.067 |
| Pressure adjustment | -12 | (-26, -9) | 0 | (-19, 0) | 11.3 | (2.1, 20.4) | 0.016 |
| Removal of retractors | -8 | (-10, -3) | -6 | (-9, -4) | 1.1 | (-1.7, 3.9) | 0.436 |

Data are summarised as median (25th–75th percentages, IQR).

*P* value by generalized estimating equation models

CI, confidence interval; SD, standard deviation
